# A one-dimensional coordination polymer, *catena*-poly[[[[*N*-ethyl-*N*-(pyridin-4-ylmeth­yl)di­thio­carbamato-κ^2^ *S*,*S*′]zinc(II)]-μ_2_-*N*-ethyl-*N*-(pyridin-4-ylmeth­yl)di­thio­carbamato-κ^3^ *S*,*S*′:*N*] 4-methyl­pyridine hemisolvate]

**DOI:** 10.1107/S2056989017010179

**Published:** 2017-07-13

**Authors:** Pavel Poplaukhin, Hadi D. Arman, Edward R. T. Tiekink

**Affiliations:** a Chemical Abstracts Service, 2540 Olentangy River Rd, Columbus, Ohio 43202, USA; b Department of Chemistry, The University of Texas at San Antonio, One UTSA Circle, San Antonio, Texas 78249-0698, USA; c Centre for Crystalline Materials, School of Science and Technology, Sunway University, 47500 Bandar Sunway, Selangor Darul Ehsan, Malaysia

**Keywords:** crystal structure, coordination polymer, zinc, di­thio­carbamate, methyl­pyridine

## Abstract

The title compound, {[Zn(C_9_H_11_N_2_S_2_)_2_]·0.5C_6_H_7_N}_*n*_, comprises two independent, but chemically similar, Zn[S_2_CN(Et)CH_2_py]_2_ residues and a 4-methyl­pyridine solvent mol­ecule in the asymmetric unit. The Zn-containing units are connected into a one-dimensional coordination polymer (zigzag topology) propagating in the [010] direction, with one di­thio­carbamate ligand bridging in a μ_2_-κ^3^ mode, employing one pyridyl N and both di­thio­carbamate S atoms, while the other is κ^2^-chelating. In each case, the resultant ZnNS_4_ coordination geometry approximates a square pyramid, with the pyridyl N atom in the apical position. In the crystal, the chains are linked into a three-dimensional architecture by methyl- and pyridyl-C—H⋯S, methyl­ene-C—H⋯N(pyrid­yl) and pyridyl-C—H⋯π(ZnS_2_C) inter­actions. The connection between the chain and the 4-methyl­pyridine solvent mol­ecule is of the type pyridyl-C—H⋯N(4-methyl­pyridine).

## Abstract 2

The title compound, {Zn[S_2_CN(Et)CH_2_py]_2_.(4-methyl­pyridine)_0.5_}_*n*_, is a one-dimensional coordination polymer with a zigzag topology.

## Chemical context   

The most recent surveys of the structural chemistry of the binary zinc-triad di­thio­carbamates, *i.e*. mol­ecules of the general formula *M*(S_2_CN*RR*′)_2_ for *M* = Zn, Cd and Hg, indicated that up to that point, *R* and *R*′ were generally restricted to alkyl groups, with only rare examples of *R* being an aryl group (Tiekink, 2003 ▸; Hogarth, 2005 ▸). However, since around that time there has been increasing inter­est in elaborating di­thio­carbamate ligands to enhance their functionality for systematic structural studies. This enhancement can be achieved in two ways utilizing their facile procedure of synthesis, *i.e*. the reaction of CS_2_ with an amine in the presence of base. Hence, the utilization of di­amines can lead to bis­(di­thio­carbamates), *e.g*. ^−^S_2_CN—*R*—CS_2_^−^, *R* = alk­yl/aryl (*e.g*. Cookson & Beer, 2007 ▸; Knight *et al.*, 2009 ▸; Oliver *et al.* 2011 ▸). Alternatively, the chosen amine can carry a functional group capable of additional coordination to a metal cation, typically a pyridyl group (*e.g*. Barba *et al.*, 2012 ▸; Singh *et al.*, 2014 ▸) or groups capable of forming hydrogen-bonding inter­actions (*e.g*. Benson *et al.*, 2007 ▸; Howie *et al.*, 2008 ▸). It is the former class of ligand with a pyridyl substituent which forms the focus of the present contribution.

Previous structural studies have revealed a diversity of coordination modes in the zinc-triad elements coordinated by di­thio­carbamate ligands functionalized with pyridyl substituents. Thus, a two-dimensional architecture is found in centrosymmetric {Zn[S_2_CN(CH_2_ferrocen­yl)CH_2_py]_2_}_*n*_, with both pyridyl N atoms being coordinating (Kumar *et al.*, 2016 ▸). In the cadmium analogue, isolated as a 1,10-phenanthroline (phen) adduct, *i.e*. Cd[S_2_CN(CH_2_ferrocen­yl)CH_2_py]_2_(phen), no additional Cd—N(pyrid­yl) inter­actions are formed in the crystal as the cadmium cation is coordinatively saturated (Kumar *et al.*, 2016 ▸). However, in {Cd{[S_2_CN(CH_2_Ph)CH_2_py]_2_}_*n*_ and related species, all potential donor atoms are coordinating, leading to a two-dimensional coordination polymer (Kumar *et al.*, 2014 ▸). It is inter­esting to note that zero-dimensional aggregation can also occur, as in the case of {Cd[S_2_CN(1H-indol-3-ylmeth­yl)CH_2_(CH_2_py)]_2_}_2_, where the tridentate mode of coordination of one di­thio­carbamate is retained, but aggregation leads to a dimer only (Kumar *et al.*, 2014 ▸). This may be a result of the now well established steric effects in 1,1-di­thiol­ate chemistry (Tiekink, 2003 ▸, 2006 ▸). Several related structures are also available for mercury. In {Hg[S_2_CN(CH_2_Py)_2_]_2_]}_*n*_, with two pyridyl groups per di­thio­carbamate ligand, an unusual one-dimensional coordination polymer with a twisted topology is found in the crystal, as one pyridyl N atom is noncoordinating (Yadav *et al.*, 2014 ▸; Jotani *et al.*, 2016 ▸). When one CH_2_py group is replaced by a methyl substitutent, as in {Hg[S_2_CN(Me)CH_2_Py]_2_}_*n*_ (Singh *et al.*, 2014 ▸), a one-dimensional coordination polymer is also found. Again, when one substituent is large, *i.e*. as in {Hg[S_2_CN{CH_2_(1-methyl-1*H*-pyrrol-2-yl)}CH_2_Py]_2_}_*n*_ (Yadav *et al.*, 2014 ▸), no Hg—N(pyrid­yl) inter­actions are found. Very recently, the crystal structure of a binary compound, isolated as the 3-methyl­pyridine monosolvate, *i.e*. {Cd[S_2_CN(Et)CH_2_py]_2_·3-methyl­pyridine}_*n*_, was described and found to feature two *S*,*S*′,*N*-tridentate di­thio­carbamate ligands, leading to a two-dimensional coordination polymer (Arman *et al.*, 2017 ▸), as seen earlier in some of the precedents mentioned above (Kumar *et al.*, 2014 ▸); the 3-methyl­pyridine solvent mol­ecules reside in square-shaped channels. In continuation of these structural studies, herein, the crystallographic characterization of a closely related zinc compound to the last mentioned species, namely {Zn[S_2_CN(Et)CH_2_py]_2_·(4-methyl­pyri­dine)_0.5_}_*n*_, is described.
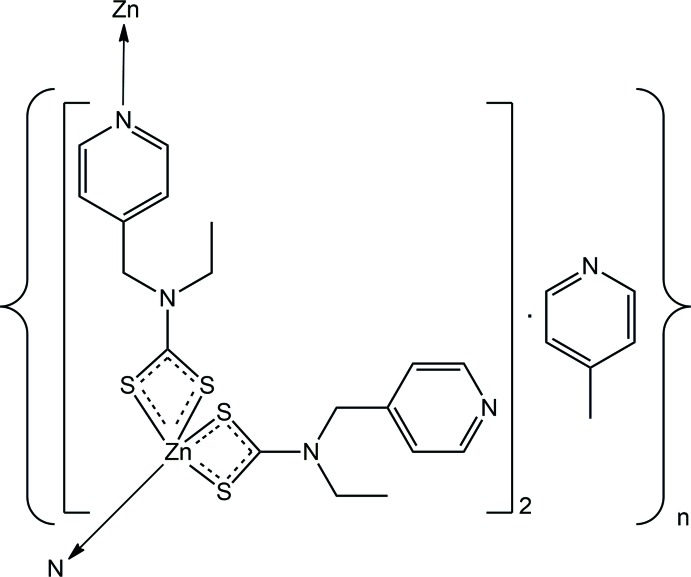


## Structural commentary   

**Figure 1:**
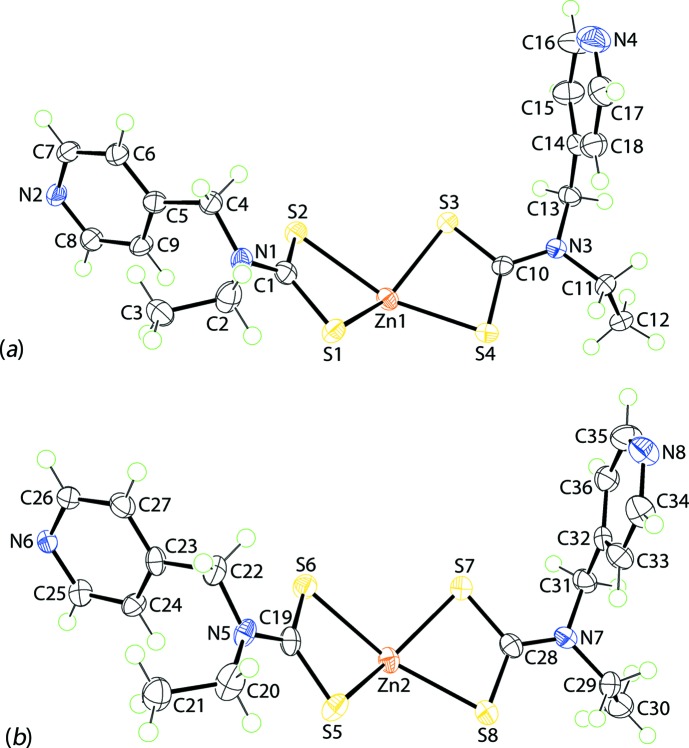
The mol­ecular structures of the two independent Zn[S_2_CN(Et)CH_2_py]_2_ fragments in the asymmetric unit of (I), showing the atom-labelling scheme and displacement ellipsoids at the 50% probability level.

**Figure 2:**
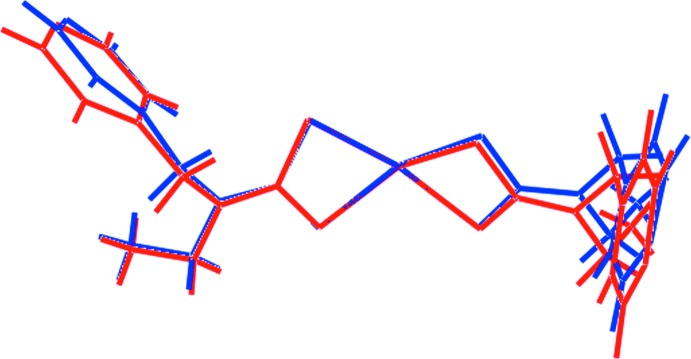
A mol­ecular overlay diagram of the two independent mol­ecules of Zn[S_2_CN(Et)CH_2_py]_2_. The Zn1-containing mol­ecule is shown in red and the mol­ecules have been overlapped so that the two more symmetrically chelating di­thio­carbamate ligands are coincident.

The asymmetric unit of (I) comprises two independent Zn[S_2_CN(Et)CH_2_py]_2_ residues, shown in Fig. 1 ▸, and a 4-methyl­pyridine solvent mol­ecule. Each of the di­thio­carbamate ligands is chelating, forming approximately similar Zn—S bond lengths, see data in Table 1 ▸. For the Zn1-containing mol­ecule, the disparity in the Zn—S bond lengths, *i.e*. Δ(Zn—S) = [Zn—S(long) − Zn—S(short)], for the S1-di­thio­carbamate ligand of 0.32 Å is greater than the value of 0.10 Å for the S3-di­thio­carbamate ligand. For the Zn2-mol­ecule, these differences diminish to 0.23 and 0.09 Å for the S5- and S7-di­thio­carbamate ligands, respectively. The similarity of the structures is emphasized in the overlay diagram of Fig. 2 ▸, showing minor variations in the orientations of the pyridyl rings and in the relationship between the two chelate rings. In each of the Zn-containing mol­ecules, one di­thio­carbamate ligand coordinates in a μ_2_-κ^3^ mode, chelating one Zn^II^ cation and simultaneously bridging another *via* the pyridyl N atom. It is noted that it is the di­thio­carbamate ligand that forms the more equivalent Zn—S bond lengths in each residue that forms the bridging inter­actions. The resultant coordination geometry for each Zn^II^ cation is based on an NS_4_ donor set.

For five-coordinate species, the value computed for τ is a useful indicator of the adopted coordination geometry, with the values of τ ranging from 0 to 1 for ideal square-pyramidal and trigonal–bipyramidal geometries, respectively (Addison *et al.*, 1984 ▸). In (I), the values of τ for Zn1 and Zn2 are 0.33 and 0.23, respectively, indicating that Zn2 is closer to a square pyramid than Zn1. In each case, the pyridyl N atom occupies the approximately apical position, as indicated by the range of N—Zn1—S angles of 97.62 (8)–111.76 (9)° and the narrower range of N—Zn2—S angles of 99.72 (9)–110.48 (9)°. In this description, the Zn1 cation lies 0.6827 (6) Å above the best plane through the four S atoms, *i.e.* S1–S4 (r.m.s. deviation = 0.1721 Å), in the direction of the pyridyl N6 atom. For the Zn2-mol­ecule, the deviation of the Zn2 cation from the S_4_ plane is 0.6018 (6) Å and the r.m.s. deviation through the S5–S8 atoms is 0.1273 Å.

**Figure 3:**
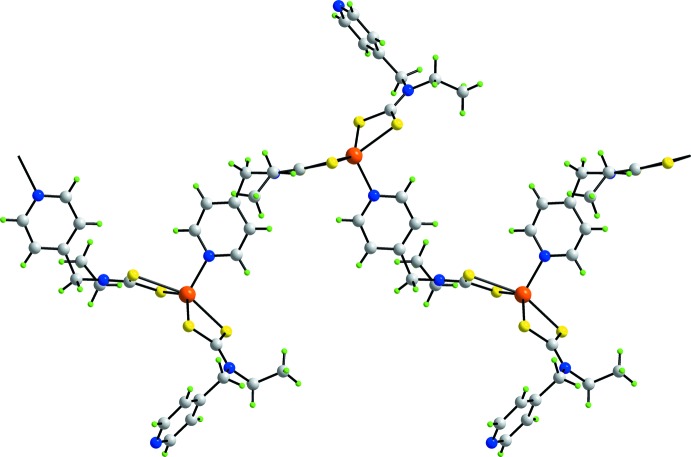
The one-dimensional coordination polymer in (I), aligned along [010].

The result of the presence of equal numbers of chelating and bridging ligands in (I) is the formation of a supra­molecular polymer aligned along [010], as illustrated in Fig. 3 ▸. The topology of the chain is zigzag. Finally, the 4-methyl­pyridine solvent mol­ecule is non-coordinating.

The most closely related structure in the literature for comparison is that of the aforementioned recently reported {Cd[S_2_CN(Et)CH_2_py]_2_·3-methyl­pyridine}_*n*_, which was also isolated from an experiment attempting to coordinate isomeric methyl­pyridines to the heavy element (Arman *et al.*, 2017 ▸). The crucial difference between the two structures is that in the cadmium crystal, both di­thio­carbamates adopt a μ_2_-κ^3^ coordination mode, leading to a *cis*-N_2_S_4_ coordination geometry and a two-dimensional framework with a flat topology. It is highly likely that the disparity in supra­molecular aggregation in the zinc and cadmium compounds arises from the greater ability of the larger Cd atom to expand its donor set.

## Supra­molecular features   

**Figure 4:**
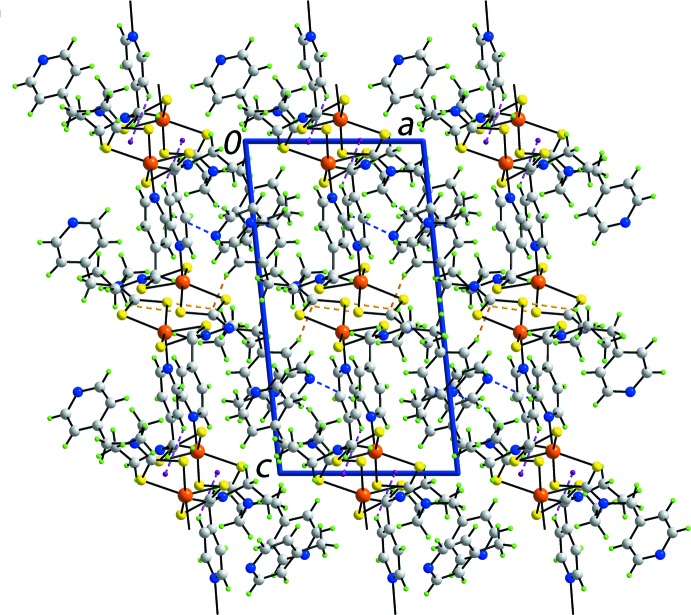
A view of the unit-cell contents in projection down the *b* axis. The C—H⋯S, C—H⋯N and C—H⋯π inter­actions are shown in orange, blue and purple dashed lines, respectively.

As mentioned above, the supra­molecular chains in the crystal of (I) are aligned along [010]. In the crystal, these chains are connected into a three-dimensional architecture by a number of weak inter­molecular inter­actions, as summarized in Table 2 ▸. There are two distinct C—H⋯S inter­actions, with the donors being methyl- and pyridyl-C—H groups, as well as a methyl­ene-C—H⋯N(pyrid­yl) inter­action. The other connection between chains is of the type pyridyl-C—H⋯π(Zn1,S3,S4,C10), an inter­action well known in metal di­thio­carbamates (Tiekink & Zukerman-Schpector, 2011 ▸) and, indeed, other metal systems (Tiekink, 2017 ▸). The main connection identified between the 4-methyl­pyridine solvent mol­ecule and the chain is of the type pyridyl-C—H⋯N(4-methyl­pyridine). An illustration of the mol­ecular packing is given in Fig. 4 ▸.

## Database survey   

The di­thio­carabmate anion, ^−^[S_2_CN(Et)CH_2_py], found in (I) and in {Cd[S_2_CN(Et)CH_2_py]_2_·3-methyl­pyridine}_*n*_ (Arman *et al.*, 2017 ▸), has been structurally characterized in its free form, *i.e*. as its potassium 1,4,7,10,13,16-hexa­oxa­cyclo­octa­decane (*i.e*. 18-crown-6) salt (Arman *et al.*, 2013 ▸). The pyridyl N atom is noncoordinating in this structure, the K^+^ ion being connected to S and O atoms only, within an O_6_S_2_ donor set. There is also a series of three diorganotin structures with this di­thio­carbamate ligand, *i.e*. of the general formula *R*_2_Sn[S_2_CN(Et)CH_2_py]_2_, for *R* = Me, *n*Bu and Ph (Barba *et al.*, 2012 ▸). In only the *R* = Me compound is there a weak inter­molecular Sn⋯N(pyrid­yl) inter­action of 2.98 Å between the two mol­ecules comprising the asymmetric unit. This result is consistent with surveys of diorganotin bis­(di­thio­carbamate)s in general (Tiekink, 2008 ▸) which suggest that the Sn atom in these compounds does not usually increase its coordination number by forming secondary bonding inter­actions (Tiekink, 2017 ▸). Specifically, for di­methyl­tin compounds, *R*_2_Sn(S_2_CN*R*′*R*′′)_2_, a recent survey indicated that secondary bonding inter­actions occur in only 10% of their crystal structures (Zaldi *et al.*, 2017 ▸)

## Synthesis and crystallization   

The title compound was isolated from the recrystallization of Zn{[S_2_CN(Et)CH_2_py]_2_ (generated from the reaction of Zn(NO_3_)_2_·H_2_O and ^−^[S_2_CN(Et)CH_2_py]) from 4-picoline. Suitable single crystals formed upon slow evaporation of the solvent (m.p. 337–339 K).

## Refinement details   

Crystal data, data collection and structure refinement details are summarized in Table 3 ▸. The carbon-bound H atoms were placed in calculated positions (C—H = 0.95–0.99 Å) and were included in the refinement in the riding-model approximation, with *U*_iso_(H) values set at 1.2–1.5*U*_eq_(C).

## Supplementary Material

Crystal structure: contains datablock(s) I, global. DOI: 10.1107/S2056989017010179/hb7691sup1.cif

Structure factors: contains datablock(s) I. DOI: 10.1107/S2056989017010179/hb7691Isup2.hkl

CCDC reference: 1561011

Additional supporting information:  crystallographic information; 3D view; checkCIF report

## Figures and Tables

**Table 1:** Selected bond lengths (Å)

Zn1—N6	2.050 (3)	Zn2—N2^i^	2.074 (3)
Zn1—S1	2.3510 (11)	Zn2—S5	2.3723 (11)
Zn1—S2	2.6741 (11)	Zn2—S6	2.5783 (12)
Zn1—S3	2.3962 (11)	Zn2—S7	2.4036 (11)
Zn1—S4	2.4972 (11)	Zn2—S8	2.4917 (12)

Symmetry code: (i) 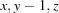.

**Table 2:** Hydrogen-bond geometry (Å, °) *Cg*1 is the ring centroid of the Zn1/S3/S4/C10 ring.

*D*—H⋯*A*	*D*—H	H⋯*A*	*D*⋯*A*	*D*—H⋯*A*
C11—H11*B*⋯N8^ii^	0.99	2.41	3.197 (5)	136
C30—H30*C*⋯S8^iii^	0.98	2.86	3.433 (5)	118
C36—H36⋯S5^iv^	0.95	2.87	3.773 (4)	158
C6—H6⋯*Cg*1^v^	0.95	2.91	3.708 (4)	142
C26—H26⋯N9^vi^	0.95	2.61	3.256 (5)	126

Symmetry codes: (ii) 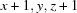; (iii) 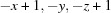; (iv) 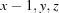; (v) 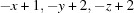; (vi) 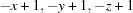.

**Table 3:** Experimental details

Crystal data	
Chemical formula	[Zn(C_9_H_11_N_2_S_2_)_2_]·0.5C_6_H_7_N
*M*_r_	534.57
Crystal system, space group	Triclinic, *P* 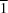
Temperature (K)	98
*a*, *b*, *c* (Å)	9.419 (2), 15.299 (4), 17.149 (4)
α, β, γ (°)	88.871 (9), 83.914 (8), 75.766 (6)
*V* (Å^3^)	2381.8 (10)
*Z*	4
Radiation type	Mo *K*α
μ (mm^−1^)	1.40
Crystal size (mm)	0.30 × 0.20 × 0.08

Data collection	
Diffractometer	AFC12K/SATURN724
Absorption correction	Multi-scan (*ABSCOR*; Higashi, 1995 ▸)
*T*_min_, *T*_max_	0.549, 1
No. of measured, independent and observed [*I* > 2σ(*I*)] reflections	13748, 9827, 8634
*R*_int_	0.037

Refinement	
*R*[*F* ^2^ > 2σ(*F* ^2^)], *wR*(*F* ^2^), *S*	0.053, 0.120, 1.14
No. of reflections	9827
No. of parameters	555
H-atom treatment	H-atom parameters constrained
Δρ_max_, Δρ_min_ (e Å^−3^)	0.55, −0.81

Computer programs: *CrystalClear* (Molecular Structure Corporation & Rigaku, 2005 ▸), *SHELXS* (Sheldrick, 2008 ▸), *SHELXL2014* (Sheldrick, 2015 ▸), *ORTEP-3 for Windows* (Farrugia, 2012 ▸), *DIAMOND* (Brandenburg, 2006 ▸) and *publCIF* (Westrip, 2010 ▸).

## supplementary crystallographic information

### Crystal data

**Table:**

[Zn(C_9_H_11_N_2_S_2_)_2_]·0.5C_6_H_7_N	*Z* = 4
*M**_r_* = 534.57	*F*(000) = 1108
Triclinic, *P*1	*D*_x_ = 1.491 Mg m^−^^3^
*a* = 9.419 (2) Å	Mo *K*α radiation, λ = 0.71069 Å
*b* = 15.299 (4) Å	Cell parameters from 10781 reflections
*c* = 17.149 (4) Å	θ = 2.2–40.7°
α = 88.871 (9)°	µ = 1.40 mm^−^^1^
β = 83.914 (8)°	*T* = 98 K
γ = 75.766 (6)°	Block, colourless
*V* = 2381.8 (10) Å^3^	0.30 × 0.20 × 0.08 mm

### Data collection

**Table:**

AFC12K/SATURN724 diffractometer	9827 independent reflections
Radiation source: fine-focus sealed tube	8634 reflections with *I* > 2σ(*I*)
Graphite monochromator	*R*_int_ = 0.037
ω scans	θ_max_ = 26.5°, θ_min_ = 2.2°
Absorption correction: multi-scan (ABSCOR; Higashi, 1995)	*h* = −11→11
*T*_min_ = 0.549, *T*_max_ = 1	*k* = −19→18
13748 measured reflections	*l* = −21→21

### Refinement

**Table:**

Refinement on *F*^2^	0 restraints
Least-squares matrix: full	Hydrogen site location: inferred from neighbouring sites
*R*[*F*^2^ > 2σ(*F*^2^)] = 0.053	H-atom parameters constrained
*wR*(*F*^2^) = 0.120	*w* = 1/[σ^2^(*F*_o_^2^) + (0.0424*P*)^2^ + 1.244*P*] where *P* = (*F*_o_^2^ + 2*F*_c_^2^)/3
*S* = 1.14	(Δ/σ)_max_ = 0.001
9827 reflections	Δρ_max_ = 0.55 e Å^−^^3^
555 parameters	Δρ_min_ = −0.81 e Å^−^^3^

### Special details

**Table:**

Geometry. All esds (except the esd in the dihedral angle between two l.s. planes) are estimated using the full covariance matrix. The cell esds are taken into account individually in the estimation of esds in distances, angles and torsion angles; correlations between esds in cell parameters are only used when they are defined by crystal symmetry. An approximate (isotropic) treatment of cell esds is used for estimating esds involving l.s. planes.

### Fractional atomic coordinates and isotropic or equivalent isotropic displacement parameters (Å^2^)

**Table:**

	*x*	*y*	*z*	*U*_iso_*/*U*_eq_	
Zn1	0.54704 (5)	0.72964 (3)	0.93393 (2)	0.01760 (11)	
Zn2	0.43549 (5)	0.25774 (3)	0.57576 (2)	0.01760 (11)	
S1	0.32468 (10)	0.83898 (6)	0.96346 (5)	0.01963 (19)	
S2	0.58234 (10)	0.88714 (6)	0.87543 (5)	0.01821 (18)	
S3	0.78618 (10)	0.68982 (6)	0.97969 (5)	0.01919 (19)	
S4	0.53708 (10)	0.61206 (6)	1.03606 (5)	0.01912 (19)	
S5	0.67047 (10)	0.28992 (6)	0.55549 (5)	0.0212 (2)	
S6	0.40191 (10)	0.42263 (6)	0.61908 (5)	0.01971 (19)	
S7	0.20336 (10)	0.27671 (6)	0.52307 (5)	0.02089 (19)	
S8	0.46521 (10)	0.12755 (6)	0.48528 (5)	0.0217 (2)	
N1	0.3378 (3)	1.0087 (2)	0.92953 (16)	0.0176 (6)	
N2	0.4298 (3)	1.19659 (19)	0.68468 (17)	0.0187 (6)	
N3	0.8009 (3)	0.56341 (19)	1.09051 (17)	0.0173 (6)	
N4	1.0896 (4)	0.7366 (3)	1.2448 (2)	0.0345 (8)	
N5	0.6620 (3)	0.46253 (19)	0.58467 (17)	0.0178 (6)	
N6	0.5447 (3)	0.66729 (19)	0.82948 (16)	0.0162 (6)	
N7	0.2023 (3)	0.1338 (2)	0.43693 (17)	0.0198 (6)	
N8	−0.0182 (4)	0.3770 (2)	0.24530 (18)	0.0260 (7)	
N9	0.7781 (4)	0.1256 (2)	0.2852 (2)	0.0318 (8)	
C1	0.4081 (4)	0.9211 (2)	0.92226 (19)	0.0175 (7)	
C2	0.1861 (4)	1.0407 (3)	0.9680 (2)	0.0258 (8)	
H2A	0.1900	1.0664	1.0200	0.031*	
H2B	0.1414	0.9886	0.9764	0.031*	
C3	0.0895 (4)	1.1112 (3)	0.9207 (2)	0.0242 (8)	
H3A	−0.0104	1.1283	0.9479	0.036*	
H3B	0.0862	1.0865	0.8688	0.036*	
H3C	0.1299	1.1644	0.9148	0.036*	
C4	0.4188 (4)	1.0786 (2)	0.91370 (19)	0.0186 (7)	
H4A	0.5225	1.0528	0.9235	0.022*	
H4B	0.3775	1.1282	0.9520	0.022*	
C5	0.4183 (4)	1.1186 (2)	0.8325 (2)	0.0183 (7)	
C6	0.4609 (4)	1.1986 (2)	0.8213 (2)	0.0187 (7)	
H6	0.4855	1.2282	0.8643	0.022*	
C7	0.4674 (4)	1.2355 (2)	0.7466 (2)	0.0194 (7)	
H7	0.4992	1.2897	0.7391	0.023*	
C8	0.3872 (4)	1.1200 (2)	0.6959 (2)	0.0192 (7)	
H8	0.3598	1.0928	0.6525	0.023*	
C9	0.3813 (4)	1.0782 (2)	0.76892 (19)	0.0185 (7)	
H9	0.3522	1.0230	0.7747	0.022*	
C10	0.7165 (4)	0.6157 (2)	1.04110 (19)	0.0151 (7)	
C11	0.7438 (4)	0.4998 (2)	1.1437 (2)	0.0212 (8)	
H11A	0.6378	0.5255	1.1596	0.025*	
H11B	0.7957	0.4922	1.1916	0.025*	
C12	0.7640 (4)	0.4080 (3)	1.1055 (2)	0.0248 (8)	
H12A	0.7334	0.3664	1.1441	0.037*	
H12B	0.8678	0.3843	1.0861	0.037*	
H12C	0.7036	0.4143	1.0615	0.037*	
C13	0.9591 (4)	0.5577 (2)	1.0897 (2)	0.0185 (7)	
H13A	0.9985	0.5688	1.0355	0.022*	
H13B	1.0092	0.4955	1.1036	0.022*	
C14	0.9978 (4)	0.6223 (2)	1.1441 (2)	0.0185 (7)	
C15	1.1310 (4)	0.6461 (3)	1.1276 (2)	0.0277 (9)	
H15	1.1941	0.6238	1.0816	0.033*	
C16	1.1711 (5)	0.7026 (3)	1.1786 (3)	0.0359 (10)	
H16	1.2623	0.7183	1.1658	0.043*	
C17	0.9631 (5)	0.7129 (3)	1.2598 (2)	0.0283 (9)	
H17	0.9029	0.7356	1.3066	0.034*	
C18	0.9122 (4)	0.6575 (3)	1.2122 (2)	0.0249 (8)	
H18	0.8197	0.6438	1.2261	0.030*	
C19	0.5864 (4)	0.3983 (2)	0.58684 (19)	0.0175 (7)	
C20	0.8195 (4)	0.4443 (3)	0.5556 (2)	0.0305 (9)	
H20A	0.8293	0.4685	0.5018	0.037*	
H20B	0.8611	0.3783	0.5526	0.037*	
C21	0.9081 (4)	0.4856 (3)	0.6068 (2)	0.0281 (9)	
H21A	1.0129	0.4660	0.5879	0.042*	
H21B	0.8924	0.4660	0.6611	0.042*	
H21C	0.8761	0.5515	0.6044	0.042*	
C22	0.5849 (4)	0.5575 (2)	0.5968 (2)	0.0202 (8)	
H22A	0.4857	0.5667	0.5789	0.024*	
H22B	0.6388	0.5945	0.5629	0.024*	
C23	0.5673 (4)	0.5923 (2)	0.68008 (19)	0.0170 (7)	
C24	0.6113 (4)	0.5384 (2)	0.7445 (2)	0.0181 (7)	
H24	0.6492	0.4751	0.7381	0.022*	
C25	0.5989 (4)	0.5782 (2)	0.8170 (2)	0.0184 (7)	
H25	0.6300	0.5411	0.8601	0.022*	
C26	0.4962 (4)	0.7180 (2)	0.7679 (2)	0.0189 (7)	
H26	0.4533	0.7806	0.7762	0.023*	
C27	0.5060 (4)	0.6832 (2)	0.6938 (2)	0.0205 (8)	
H27	0.4707	0.7215	0.6521	0.025*	
C28	0.2825 (4)	0.1734 (2)	0.4770 (2)	0.0182 (7)	
C29	0.2630 (4)	0.0457 (2)	0.3968 (2)	0.0241 (8)	
H29A	0.3719	0.0314	0.3930	0.029*	
H29B	0.2337	0.0499	0.3428	0.029*	
C30	0.2093 (5)	−0.0302 (3)	0.4398 (3)	0.0323 (10)	
H30A	0.2525	−0.0872	0.4115	0.048*	
H30B	0.1016	−0.0172	0.4423	0.048*	
H30C	0.2390	−0.0351	0.4930	0.048*	
C31	0.0470 (4)	0.1766 (2)	0.4283 (2)	0.0213 (8)	
H31A	−0.0014	0.2049	0.4788	0.026*	
H31B	−0.0031	0.1296	0.4156	0.026*	
C32	0.0291 (4)	0.2475 (2)	0.36478 (19)	0.0167 (7)	
C33	0.1391 (4)	0.2508 (3)	0.3047 (2)	0.0229 (8)	
H33	0.2329	0.2094	0.3032	0.027*	
C34	0.1093 (4)	0.3154 (3)	0.2470 (2)	0.0264 (9)	
H34	0.1849	0.3159	0.2058	0.032*	
C35	−0.1227 (4)	0.3728 (3)	0.3038 (2)	0.0275 (9)	
H35	−0.2148	0.4157	0.3042	0.033*	
C36	−0.1056 (4)	0.3105 (3)	0.3632 (2)	0.0231 (8)	
H36	−0.1845	0.3105	0.4027	0.028*	
C37	0.8173 (4)	0.0465 (3)	0.3202 (2)	0.0282 (9)	
H37	0.7525	0.0328	0.3624	0.034*	
C38	0.9486 (4)	−0.0178 (3)	0.2986 (2)	0.0288 (9)	
H38	0.9703	−0.0742	0.3245	0.035*	
C39	1.0473 (4)	0.0021 (3)	0.2383 (2)	0.0269 (8)	
C40	1.0051 (5)	0.0859 (3)	0.2018 (2)	0.0322 (9)	
H40	1.0677	0.1027	0.1602	0.039*	
C41	0.8712 (5)	0.1440 (3)	0.2271 (3)	0.0353 (10)	
H41	0.8444	0.2004	0.2015	0.042*	
C42	1.1917 (5)	−0.0633 (3)	0.2133 (3)	0.0387 (11)	
H42A	1.2049	−0.1155	0.2482	0.058*	
H42B	1.2722	−0.0337	0.2161	0.058*	
H42C	1.1921	−0.0833	0.1593	0.058*	

### Atomic displacement parameters (Å^2^)

**Table:**

	*U*^11^	*U*^22^	*U*^33^	*U*^12^	*U*^13^	*U*^23^
Zn1	0.0182 (2)	0.0187 (2)	0.0157 (2)	−0.00322 (16)	−0.00350 (16)	−0.00183 (16)
Zn2	0.0214 (2)	0.0196 (2)	0.0143 (2)	−0.00902 (17)	−0.00376 (16)	0.00265 (16)
S1	0.0195 (5)	0.0172 (4)	0.0221 (5)	−0.0057 (3)	0.0010 (3)	0.0019 (3)
S2	0.0184 (4)	0.0189 (4)	0.0176 (4)	−0.0056 (3)	−0.0010 (3)	0.0013 (3)
S3	0.0194 (5)	0.0199 (4)	0.0201 (4)	−0.0077 (4)	−0.0040 (3)	0.0040 (3)
S4	0.0170 (4)	0.0214 (5)	0.0211 (4)	−0.0079 (3)	−0.0044 (3)	0.0017 (3)
S5	0.0227 (5)	0.0166 (4)	0.0240 (5)	−0.0072 (4)	0.0047 (4)	−0.0042 (3)
S6	0.0192 (5)	0.0188 (4)	0.0213 (4)	−0.0052 (3)	−0.0014 (3)	−0.0017 (3)
S7	0.0222 (5)	0.0199 (4)	0.0208 (5)	−0.0040 (4)	−0.0051 (4)	−0.0024 (3)
S8	0.0195 (5)	0.0237 (5)	0.0222 (5)	−0.0039 (4)	−0.0059 (3)	−0.0023 (4)
N1	0.0176 (16)	0.0186 (15)	0.0157 (15)	−0.0037 (12)	−0.0003 (11)	0.0021 (12)
N2	0.0240 (17)	0.0169 (15)	0.0171 (15)	−0.0075 (12)	−0.0049 (12)	0.0042 (12)
N3	0.0167 (15)	0.0166 (15)	0.0200 (15)	−0.0055 (12)	−0.0051 (12)	0.0022 (12)
N4	0.038 (2)	0.038 (2)	0.032 (2)	−0.0151 (17)	−0.0091 (16)	−0.0055 (16)
N5	0.0215 (16)	0.0153 (15)	0.0185 (15)	−0.0095 (12)	0.0012 (12)	−0.0023 (12)
N6	0.0150 (15)	0.0174 (15)	0.0168 (15)	−0.0042 (12)	−0.0038 (11)	−0.0019 (11)
N7	0.0173 (16)	0.0196 (16)	0.0224 (16)	−0.0027 (12)	−0.0062 (12)	−0.0001 (12)
N8	0.0211 (17)	0.0340 (19)	0.0215 (17)	−0.0042 (14)	−0.0028 (13)	0.0042 (14)
N9	0.028 (2)	0.0290 (19)	0.039 (2)	−0.0058 (15)	−0.0105 (16)	−0.0053 (16)
C1	0.0194 (18)	0.0211 (18)	0.0123 (16)	−0.0049 (14)	−0.0026 (13)	−0.0005 (13)
C2	0.022 (2)	0.024 (2)	0.028 (2)	−0.0031 (16)	0.0052 (16)	0.0058 (16)
C3	0.020 (2)	0.027 (2)	0.026 (2)	−0.0041 (15)	−0.0064 (15)	0.0005 (16)
C4	0.0234 (19)	0.0183 (18)	0.0155 (17)	−0.0070 (14)	−0.0042 (14)	−0.0003 (14)
C5	0.0152 (18)	0.0196 (18)	0.0203 (18)	−0.0050 (14)	−0.0009 (13)	0.0013 (14)
C6	0.0215 (19)	0.0168 (17)	0.0190 (18)	−0.0061 (14)	−0.0036 (14)	−0.0003 (14)
C7	0.0222 (19)	0.0175 (18)	0.0207 (18)	−0.0092 (14)	−0.0030 (14)	0.0022 (14)
C8	0.0225 (19)	0.0207 (18)	0.0153 (17)	−0.0064 (15)	−0.0033 (14)	−0.0001 (14)
C9	0.025 (2)	0.0180 (17)	0.0153 (17)	−0.0103 (15)	−0.0035 (14)	−0.0002 (14)
C10	0.0172 (17)	0.0121 (16)	0.0164 (17)	−0.0040 (13)	−0.0016 (13)	−0.0030 (13)
C11	0.0202 (19)	0.0246 (19)	0.0213 (19)	−0.0107 (15)	−0.0034 (14)	0.0083 (15)
C12	0.024 (2)	0.024 (2)	0.031 (2)	−0.0141 (16)	−0.0082 (16)	0.0081 (16)
C13	0.0141 (17)	0.0193 (18)	0.0216 (18)	−0.0024 (14)	−0.0031 (14)	−0.0004 (14)
C14	0.0183 (18)	0.0182 (17)	0.0200 (18)	−0.0044 (14)	−0.0076 (14)	0.0041 (14)
C15	0.023 (2)	0.030 (2)	0.030 (2)	−0.0088 (17)	0.0029 (16)	−0.0066 (17)
C16	0.028 (2)	0.045 (3)	0.041 (3)	−0.020 (2)	−0.0033 (19)	−0.008 (2)
C17	0.031 (2)	0.031 (2)	0.023 (2)	−0.0057 (17)	−0.0042 (16)	−0.0016 (16)
C18	0.023 (2)	0.024 (2)	0.028 (2)	−0.0068 (16)	−0.0029 (16)	0.0010 (16)
C19	0.0235 (19)	0.0225 (18)	0.0086 (16)	−0.0096 (15)	−0.0014 (13)	0.0000 (13)
C20	0.026 (2)	0.034 (2)	0.034 (2)	−0.0154 (18)	0.0086 (17)	−0.0108 (18)
C21	0.028 (2)	0.029 (2)	0.030 (2)	−0.0108 (17)	−0.0022 (17)	−0.0037 (17)
C22	0.032 (2)	0.0171 (18)	0.0138 (17)	−0.0101 (15)	−0.0033 (14)	0.0015 (14)
C23	0.0177 (18)	0.0224 (18)	0.0141 (17)	−0.0111 (14)	−0.0007 (13)	−0.0010 (14)
C24	0.0208 (19)	0.0135 (17)	0.0199 (18)	−0.0041 (14)	−0.0019 (14)	0.0006 (14)
C25	0.0225 (19)	0.0192 (18)	0.0159 (17)	−0.0096 (15)	−0.0020 (14)	0.0034 (14)
C26	0.0204 (19)	0.0157 (17)	0.0208 (18)	−0.0033 (14)	−0.0068 (14)	0.0010 (14)
C27	0.0215 (19)	0.0202 (18)	0.0210 (18)	−0.0057 (15)	−0.0072 (14)	0.0039 (15)
C28	0.0196 (18)	0.0202 (18)	0.0166 (17)	−0.0076 (14)	−0.0043 (14)	0.0049 (14)
C29	0.025 (2)	0.0213 (19)	0.028 (2)	−0.0065 (16)	−0.0086 (16)	−0.0061 (16)
C30	0.033 (2)	0.020 (2)	0.047 (3)	−0.0070 (17)	−0.0152 (19)	0.0016 (18)
C31	0.0170 (18)	0.0229 (19)	0.0258 (19)	−0.0067 (15)	−0.0058 (14)	0.0007 (15)
C32	0.0191 (18)	0.0188 (17)	0.0133 (16)	−0.0059 (14)	−0.0041 (13)	−0.0020 (13)
C33	0.0158 (18)	0.028 (2)	0.026 (2)	−0.0065 (15)	−0.0048 (14)	0.0016 (16)
C34	0.020 (2)	0.038 (2)	0.023 (2)	−0.0103 (17)	−0.0006 (15)	0.0019 (17)
C35	0.023 (2)	0.030 (2)	0.024 (2)	0.0010 (16)	0.0029 (16)	0.0011 (16)
C36	0.022 (2)	0.028 (2)	0.0168 (18)	−0.0025 (16)	−0.0002 (14)	−0.0025 (15)
C37	0.024 (2)	0.040 (2)	0.025 (2)	−0.0143 (18)	−0.0054 (16)	−0.0079 (17)
C38	0.025 (2)	0.033 (2)	0.034 (2)	−0.0124 (17)	−0.0104 (17)	−0.0052 (18)
C39	0.021 (2)	0.030 (2)	0.033 (2)	−0.0088 (16)	−0.0095 (16)	−0.0062 (17)
C40	0.033 (2)	0.036 (2)	0.028 (2)	−0.0092 (19)	−0.0010 (17)	−0.0058 (18)
C41	0.040 (3)	0.032 (2)	0.036 (2)	−0.009 (2)	−0.014 (2)	0.0038 (19)
C42	0.028 (2)	0.034 (2)	0.054 (3)	−0.0081 (19)	−0.001 (2)	−0.013 (2)

### Geometric parameters (Å, º)

**Table:**

Zn1—N6	2.050 (3)	C12—H12A	0.9800
Zn1—S1	2.3510 (11)	C12—H12B	0.9800
Zn1—S2	2.6741 (11)	C12—H12C	0.9800
Zn1—S3	2.3962 (11)	C13—C14	1.505 (5)
Zn1—S4	2.4972 (11)	C13—H13A	0.9900
Zn2—N2^i^	2.074 (3)	C13—H13B	0.9900
Zn2—S5	2.3723 (11)	C14—C18	1.384 (5)
Zn2—S6	2.5783 (12)	C14—C15	1.391 (5)
Zn2—S7	2.4036 (11)	C15—C16	1.384 (6)
Zn2—S8	2.4917 (12)	C15—H15	0.9500
S1—C1	1.740 (4)	C16—H16	0.9500
S2—C1	1.710 (4)	C17—C18	1.389 (5)
S3—C10	1.732 (3)	C17—H17	0.9500
S4—C10	1.715 (3)	C18—H18	0.9500
S5—C19	1.721 (4)	C20—C21	1.518 (5)
S6—C19	1.718 (4)	C20—H20A	0.9900
S7—C28	1.738 (4)	C20—H20B	0.9900
S8—C28	1.711 (4)	C21—H21A	0.9800
N1—C1	1.342 (4)	C21—H21B	0.9800
N1—C4	1.465 (4)	C21—H21C	0.9800
N1—C2	1.478 (5)	C22—C23	1.513 (5)
N2—C8	1.333 (4)	C22—H22A	0.9900
N2—C7	1.348 (5)	C22—H22B	0.9900
N2—Zn2^ii^	2.074 (3)	C23—C27	1.383 (5)
N3—C10	1.339 (4)	C23—C24	1.402 (5)
N3—C13	1.469 (4)	C24—C25	1.377 (5)
N3—C11	1.479 (4)	C24—H24	0.9500
N4—C17	1.328 (5)	C25—H25	0.9500
N4—C16	1.339 (6)	C26—C27	1.372 (5)
N5—C19	1.345 (4)	C26—H26	0.9500
N5—C22	1.463 (4)	C27—H27	0.9500
N5—C20	1.475 (5)	C29—C30	1.525 (5)
N6—C26	1.350 (4)	C29—H29A	0.9900
N6—C25	1.345 (4)	C29—H29B	0.9900
N7—C28	1.327 (4)	C30—H30A	0.9800
N7—C31	1.469 (4)	C30—H30B	0.9800
N7—C29	1.481 (5)	C30—H30C	0.9800
N8—C34	1.335 (5)	C31—C32	1.515 (5)
N8—C35	1.341 (5)	C31—H31A	0.9900
N9—C37	1.327 (5)	C31—H31B	0.9900
N9—C41	1.329 (6)	C32—C33	1.391 (5)
C2—C3	1.513 (5)	C32—C36	1.394 (5)
C2—H2A	0.9900	C33—C34	1.384 (5)
C2—H2B	0.9900	C33—H33	0.9500
C3—H3A	0.9800	C34—H34	0.9500
C3—H3B	0.9800	C35—C36	1.376 (5)
C3—H3C	0.9800	C35—H35	0.9500
C4—C5	1.510 (5)	C36—H36	0.9500
C4—H4A	0.9900	C37—C38	1.398 (6)
C4—H4B	0.9900	C37—H37	0.9500
C5—C9	1.381 (5)	C38—C39	1.396 (6)
C5—C6	1.383 (5)	C38—H38	0.9500
C6—C7	1.391 (5)	C39—C40	1.401 (6)
C6—H6	0.9500	C39—C42	1.503 (6)
C7—H7	0.9500	C40—C41	1.384 (6)
C8—C9	1.397 (5)	C40—H40	0.9500
C8—H8	0.9500	C41—H41	0.9500
C9—H9	0.9500	C42—H42A	0.9800
C11—C12	1.521 (5)	C42—H42B	0.9800
C11—H11A	0.9900	C42—H42C	0.9800
C11—H11B	0.9900		
			
N6—Zn1—S1	109.98 (8)	C18—C14—C13	124.4 (3)
N6—Zn1—S3	111.76 (9)	C15—C14—C13	118.7 (3)
S1—Zn1—S3	137.18 (4)	C16—C15—C14	119.5 (4)
N6—Zn1—S4	105.21 (8)	C16—C15—H15	120.2
S1—Zn1—S4	103.71 (4)	C14—C15—H15	120.2
S3—Zn1—S4	74.11 (3)	N4—C16—C15	124.2 (4)
N6—Zn1—S2	97.62 (8)	N4—C16—H16	117.9
S1—Zn1—S2	71.89 (3)	C15—C16—H16	117.9
S3—Zn1—S2	93.44 (3)	N4—C17—C18	124.8 (4)
S4—Zn1—S2	156.73 (3)	N4—C17—H17	117.6
N2^i^—Zn2—S5	105.06 (9)	C18—C17—H17	117.6
N2^i^—Zn2—S7	110.48 (9)	C14—C18—C17	119.2 (4)
S5—Zn2—S7	144.31 (4)	C14—C18—H18	120.4
N2^i^—Zn2—S8	101.93 (9)	C17—C18—H18	120.4
S5—Zn2—S8	102.19 (4)	N5—C19—S6	120.9 (3)
S7—Zn2—S8	73.89 (3)	N5—C19—S5	121.2 (3)
N2^i^—Zn2—S6	99.72 (9)	S6—C19—S5	117.9 (2)
S5—Zn2—S6	72.94 (3)	N5—C20—C21	113.1 (3)
S7—Zn2—S6	97.50 (3)	N5—C20—H20A	108.9
S8—Zn2—S6	158.31 (3)	C21—C20—H20A	108.9
C1—S1—Zn1	89.47 (13)	N5—C20—H20B	108.9
C1—S2—Zn1	79.95 (12)	C21—C20—H20B	108.9
C10—S3—Zn1	85.51 (12)	H20A—C20—H20B	107.8
C10—S4—Zn1	82.71 (11)	C20—C21—H21A	109.5
C19—S5—Zn2	87.66 (12)	C20—C21—H21B	109.5
C19—S6—Zn2	81.29 (12)	H21A—C21—H21B	109.5
C28—S7—Zn2	85.37 (12)	C20—C21—H21C	109.5
C28—S8—Zn2	83.19 (13)	H21A—C21—H21C	109.5
C1—N1—C4	120.4 (3)	H21B—C21—H21C	109.5
C1—N1—C2	123.2 (3)	N5—C22—C23	115.9 (3)
C4—N1—C2	115.2 (3)	N5—C22—H22A	108.3
C8—N2—C7	118.4 (3)	C23—C22—H22A	108.3
C8—N2—Zn2^ii^	121.8 (2)	N5—C22—H22B	108.3
C7—N2—Zn2^ii^	119.7 (2)	C23—C22—H22B	108.3
C10—N3—C13	122.7 (3)	H22A—C22—H22B	107.4
C10—N3—C11	121.6 (3)	C27—C23—C24	117.7 (3)
C13—N3—C11	115.4 (3)	C27—C23—C22	118.3 (3)
C17—N4—C16	115.5 (4)	C24—C23—C22	124.1 (3)
C19—N5—C22	120.7 (3)	C25—C24—C23	119.2 (3)
C19—N5—C20	122.2 (3)	C25—C24—H24	120.4
C22—N5—C20	116.1 (3)	C23—C24—H24	120.4
C26—N6—C25	117.4 (3)	N6—C25—C24	123.0 (3)
C26—N6—Zn1	119.1 (2)	N6—C25—H25	118.5
C25—N6—Zn1	123.3 (2)	C24—C25—H25	118.5
C28—N7—C31	121.5 (3)	N6—C26—C27	122.9 (3)
C28—N7—C29	122.7 (3)	N6—C26—H26	118.5
C31—N7—C29	115.8 (3)	C27—C26—H26	118.5
C34—N8—C35	115.7 (3)	C26—C27—C23	119.8 (3)
C37—N9—C41	117.2 (4)	C26—C27—H27	120.1
N1—C1—S2	121.6 (3)	C23—C27—H27	120.1
N1—C1—S1	120.2 (3)	N7—C28—S8	122.7 (3)
S2—C1—S1	118.2 (2)	N7—C28—S7	120.1 (3)
N1—C2—C3	112.9 (3)	S8—C28—S7	117.1 (2)
N1—C2—H2A	109.0	N7—C29—C30	112.2 (3)
C3—C2—H2A	109.0	N7—C29—H29A	109.2
N1—C2—H2B	109.0	C30—C29—H29A	109.2
C3—C2—H2B	109.0	N7—C29—H29B	109.2
H2A—C2—H2B	107.8	C30—C29—H29B	109.2
C2—C3—H3A	109.5	H29A—C29—H29B	107.9
C2—C3—H3B	109.5	C29—C30—H30A	109.5
H3A—C3—H3B	109.5	C29—C30—H30B	109.5
C2—C3—H3C	109.5	H30A—C30—H30B	109.5
H3A—C3—H3C	109.5	C29—C30—H30C	109.5
H3B—C3—H3C	109.5	H30A—C30—H30C	109.5
N1—C4—C5	116.8 (3)	H30B—C30—H30C	109.5
N1—C4—H4A	108.1	N7—C31—C32	112.6 (3)
C5—C4—H4A	108.1	N7—C31—H31A	109.1
N1—C4—H4B	108.1	C32—C31—H31A	109.1
C5—C4—H4B	108.1	N7—C31—H31B	109.1
H4A—C4—H4B	107.3	C32—C31—H31B	109.1
C9—C5—C6	118.7 (3)	H31A—C31—H31B	107.8
C9—C5—C4	123.3 (3)	C33—C32—C36	117.4 (3)
C6—C5—C4	117.9 (3)	C33—C32—C31	123.1 (3)
C5—C6—C7	119.4 (3)	C36—C32—C31	119.4 (3)
C5—C6—H6	120.3	C34—C33—C32	118.8 (4)
C7—C6—H6	120.3	C34—C33—H33	120.6
N2—C7—C6	121.9 (3)	C32—C33—H33	120.6
N2—C7—H7	119.1	N8—C34—C33	124.6 (4)
C6—C7—H7	119.1	N8—C34—H34	117.7
N2—C8—C9	122.8 (3)	C33—C34—H34	117.7
N2—C8—H8	118.6	N8—C35—C36	124.5 (4)
C9—C8—H8	118.6	N8—C35—H35	117.8
C5—C9—C8	118.7 (3)	C36—C35—H35	117.8
C5—C9—H9	120.6	C35—C36—C32	119.0 (3)
C8—C9—H9	120.6	C35—C36—H36	120.5
N3—C10—S4	121.8 (2)	C32—C36—H36	120.5
N3—C10—S3	120.5 (3)	N9—C37—C38	123.7 (4)
S4—C10—S3	117.67 (19)	N9—C37—H37	118.1
N3—C11—C12	112.2 (3)	C38—C37—H37	118.1
N3—C11—H11A	109.2	C39—C38—C37	119.0 (4)
C12—C11—H11A	109.2	C39—C38—H38	120.5
N3—C11—H11B	109.2	C37—C38—H38	120.5
C12—C11—H11B	109.2	C38—C39—C40	116.8 (4)
H11A—C11—H11B	107.9	C38—C39—C42	121.7 (4)
C11—C12—H12A	109.5	C40—C39—C42	121.5 (4)
C11—C12—H12B	109.5	C41—C40—C39	119.4 (4)
H12A—C12—H12B	109.5	C41—C40—H40	120.3
C11—C12—H12C	109.5	C39—C40—H40	120.3
H12A—C12—H12C	109.5	N9—C41—C40	123.9 (4)
H12B—C12—H12C	109.5	N9—C41—H41	118.1
N3—C13—C14	115.2 (3)	C40—C41—H41	118.1
N3—C13—H13A	108.5	C39—C42—H42A	109.5
C14—C13—H13A	108.5	C39—C42—H42B	109.5
N3—C13—H13B	108.5	H42A—C42—H42B	109.5
C14—C13—H13B	108.5	C39—C42—H42C	109.5
H13A—C13—H13B	107.5	H42A—C42—H42C	109.5
C18—C14—C15	116.8 (4)	H42B—C42—H42C	109.5
			
C4—N1—C1—S2	−14.0 (4)	Zn2—S6—C19—N5	175.0 (3)
C2—N1—C1—S2	178.6 (3)	Zn2—S6—C19—S5	−3.67 (17)
C4—N1—C1—S1	164.1 (2)	Zn2—S5—C19—N5	−174.7 (3)
C2—N1—C1—S1	−3.4 (5)	Zn2—S5—C19—S6	3.94 (18)
Zn1—S2—C1—N1	172.1 (3)	C19—N5—C20—C21	−136.1 (4)
Zn1—S2—C1—S1	−5.99 (17)	C22—N5—C20—C21	55.4 (4)
Zn1—S1—C1—N1	−171.4 (3)	C19—N5—C22—C23	93.2 (4)
Zn1—S1—C1—S2	6.71 (19)	C20—N5—C22—C23	−98.1 (4)
C1—N1—C2—C3	−132.8 (4)	N5—C22—C23—C27	174.9 (3)
C4—N1—C2—C3	59.1 (4)	N5—C22—C23—C24	−4.2 (5)
C1—N1—C4—C5	95.4 (4)	C27—C23—C24—C25	−3.3 (5)
C2—N1—C4—C5	−96.2 (4)	C22—C23—C24—C25	175.8 (3)
N1—C4—C5—C9	−17.5 (5)	C26—N6—C25—C24	2.4 (5)
N1—C4—C5—C6	164.2 (3)	Zn1—N6—C25—C24	−172.9 (3)
C9—C5—C6—C7	−1.0 (5)	C23—C24—C25—N6	0.7 (5)
C4—C5—C6—C7	177.4 (3)	C25—N6—C26—C27	−2.9 (5)
C8—N2—C7—C6	−0.9 (5)	Zn1—N6—C26—C27	172.6 (3)
Zn2^ii^—N2—C7—C6	177.9 (3)	N6—C26—C27—C23	0.3 (5)
C5—C6—C7—N2	1.7 (6)	C24—C23—C27—C26	2.8 (5)
C7—N2—C8—C9	−0.6 (5)	C22—C23—C27—C26	−176.3 (3)
Zn2^ii^—N2—C8—C9	−179.4 (3)	C31—N7—C28—S8	−177.6 (2)
C6—C5—C9—C8	−0.4 (5)	C29—N7—C28—S8	1.0 (5)
C4—C5—C9—C8	−178.7 (3)	C31—N7—C28—S7	1.8 (4)
N2—C8—C9—C5	1.3 (6)	C29—N7—C28—S7	−179.7 (3)
C13—N3—C10—S4	172.8 (3)	Zn2—S8—C28—N7	−174.7 (3)
C11—N3—C10—S4	−0.2 (5)	Zn2—S8—C28—S7	5.98 (17)
C13—N3—C10—S3	−7.2 (5)	Zn2—S7—C28—N7	174.5 (3)
C11—N3—C10—S3	179.8 (3)	Zn2—S7—C28—S8	−6.17 (18)
Zn1—S4—C10—N3	−180.0 (3)	C28—N7—C29—C30	106.3 (4)
Zn1—S4—C10—S3	0.04 (18)	C31—N7—C29—C30	−75.1 (4)
Zn1—S3—C10—N3	180.0 (3)	C28—N7—C31—C32	80.4 (4)
Zn1—S3—C10—S4	−0.04 (18)	C29—N7—C31—C32	−98.2 (4)
C10—N3—C11—C12	88.3 (4)	N7—C31—C32—C33	20.6 (5)
C13—N3—C11—C12	−85.2 (4)	N7—C31—C32—C36	−162.7 (3)
C10—N3—C13—C14	92.3 (4)	C36—C32—C33—C34	−0.2 (5)
C11—N3—C13—C14	−94.3 (4)	C31—C32—C33—C34	176.6 (4)
N3—C13—C14—C18	26.5 (5)	C35—N8—C34—C33	−1.2 (6)
N3—C13—C14—C15	−156.3 (3)	C32—C33—C34—N8	1.3 (6)
C18—C14—C15—C16	−0.1 (6)	C34—N8—C35—C36	0.0 (6)
C13—C14—C15—C16	−177.5 (4)	N8—C35—C36—C32	0.9 (6)
C17—N4—C16—C15	−0.2 (7)	C33—C32—C36—C35	−0.8 (5)
C14—C15—C16—N4	0.5 (7)	C31—C32—C36—C35	−177.8 (4)
C16—N4—C17—C18	−0.5 (6)	C41—N9—C37—C38	−1.0 (6)
C15—C14—C18—C17	−0.5 (5)	N9—C37—C38—C39	1.9 (6)
C13—C14—C18—C17	176.7 (3)	C37—C38—C39—C40	−1.5 (5)
N4—C17—C18—C14	0.8 (6)	C37—C38—C39—C42	178.7 (4)
C22—N5—C19—S6	−10.5 (4)	C38—C39—C40—C41	0.5 (6)
C20—N5—C19—S6	−178.5 (3)	C42—C39—C40—C41	−179.7 (4)
C22—N5—C19—S5	168.1 (2)	C37—N9—C41—C40	−0.1 (6)
C20—N5—C19—S5	0.1 (5)	C39—C40—C41—N9	0.3 (7)

Symmetry codes: (i) *x*, *y*−1, *z*; (ii) *x*, *y*+1, *z*.

### Hydrogen-bond geometry (Å, º)

*Cg*1 is the ring centroid of the Zn1/S3/S4/C10 ring.

**Table:**

*D*—H···*A*	*D*—H	H···*A*	*D*···*A*	*D*—H···*A*
C11—H11*B*···N8^iii^	0.99	2.41	3.197 (5)	136
C30—H30*C*···S8^iv^	0.98	2.86	3.433 (5)	118
C36—H36···S5^v^	0.95	2.87	3.773 (4)	158
C6—H6···*Cg*1^vi^	0.95	2.91	3.708 (4)	142
C26—H26···N9^vii^	0.95	2.61	3.256 (5)	126

Symmetry codes: (iii) *x*+1, *y*, *z*+1; (iv) −*x*+1, −*y*, −*z*+1; (v) *x*−1, *y*, *z*; (vi) −*x*+1, −*y*+2, −*z*+2; (vii) −*x*+1, −*y*+1, −*z*+1.
